# Supplementary material for: Lonicera japonica Fermented by Lactobacillus plantarum Improve Multiple Patterns Driven Osteoporosis
Source: Foods. 2024 Aug 23;13(17):2649. doi: 10.3390/foods13172649 (PMC11393950; doi:10.3390/foods13172649)

Supplementary Table S1 Tibial bone tissue morphology measurement

	Con	Mod	Hse	Mix	Fer
Tb.Ar	12.258±0.842[a]	8.132±1.083[b]	8.388±2.188[b]	10.655±2.227[ab]	11.649±1.816[a]
T.Ar	21.654±3.604[a]	20.132±2.116[a]	17.723±2.183[a]	20.008±2.104[a]	20.829±3.250[a]
Tb.Pm	300.289±59.620[a]	268.802±27.396[a]	245.301±36.003[a]	287.225±72.559[a]	325.075±49.848[a]
BV/TV	0.575±0.068[a]	0.405±0.041[b]	0.472±0.094[ab]	0.535±0.118[ab]	0.559±0.029[a]
Tb.N	8.328±0.913[a]	8.056±0.952[a]	8.348±1.265[a]	8.497±1.252[a]	9.366±0.400[a]
Tb.Sp	51.572±9.621[b]	74.646±8.872[a]	64.934±16.648[ab]	55.392±0.118[ab]	47.147±4.242[b]

Supplementary Table S2 Relative abundance of key bacteria at genus level

genus	relative abundance（%）		
	Con	Mod	Fer
Lactobacillus	13.38	8.353	14.14
unclassified_f__Oscillospiraceae	2.311	0.5857	1.592
Ruminococcus_torques_group	0.3182	0.05499	1.075
norank_f__Peptococcaceae	0.1719	0.04699	0.2045
Candidatus_Stoquefichus	0.01815	0.001602	0.04058
Family_XIII_AD3011_group	0.01014	0.001068	0.09878
Prevotellaceae_Ga6A1_group	0.2082	3.354	0.01121

Supplementary Table S3 Twenty-three metabolites were significantly increased in the Mod group and significantly restored in the Fer group.

Metabolite	FC(F/M)	P_value
propoxyphene	0.7643	0.005544

4-hydroxyestrone-2-S-glutathione	0.8794	0.04129
APC	0.9372	0.01478
butabarbital	0.9251	0.04067
glutaminylglutamine	0.9394	0.04232
gamma-Glutamyllysine	0.9491	0.001652
1-(beta-D-ribofuranosyl)-1,4-dihydronicotinamide	0.9521	0.04605
aprobarbital	0.9605	0.04698
cyanoketone	0.9697	0.000263
pheneturide	0.9618	0.002456
SM(d18:1/16:1(9Z))	0.9771	0.00343
SM(d18:2(4E,14Z)/16:0)	0.9742	0.00359
2-cis-abscisate	0.969	0.003815
L-norleucine	0.9752	0.009189
DL-norvaline	0.9751	0.0122
nervonic acid	0.9674	0.04311
ampelopsin C	0.9728	0.03717
L-hypoglycin A	0.9742	0.01877
1-O-isopentyl-3-O-octadec-2-enoyl glycerol	0.9741	0.01149
N-[5-(4,5-dihydro-1H-imidazol-2-yl)-2-hydroxy-5,6,7,8-tetrahydronaphthalen-1-yl]methanesulfonamide	0.9721	0.02492
Serotonin	0.9745	0.02561
L-cysteine	0.9712	0.03635
L-methionine	0.9842	0.005176

Supplementary Table S4 Seventeen metabolites that were significantly decreased in the Mod group and significantly restored in the Fer group

Metabolite	FC(F/M)	P_value
DG(22:6(4Z,7Z,10Z,13E,15E,19Z)-OH(17)/i-12:0/0:0)	1.9153	0.04362
3,5-dichlorosalicylic acid	1.1391	0.001432
PC(20:3(8Z,11Z,14Z)-2OH(5,6)/2:0)	1.1812	0.02072
protocatechuic acid	1.1425	0.0372
ganglioside GT3 (d18:1/20:0)	1.0672	0.002474
ganglioside GD2 (d18:1/12:0)	1.1084	0.003324
3-hydroxybenzoic acid	1.0758	0.001265
2-methoxyacetaminophen sulfate	1.0915	0.02731
perylene-1,2-dione	1.0637	0.008978
12-hydroxydodecanoylcarnitine	1.0486	0.01188

methylisocitric acid	1.0642	0.0395
indole-3-acetic acid	1.0708	0.03185
2',5'-dideoxyadenosine	1.0485	0.02164
1-methyladenosine	1.0441	0.01565
1-(2-amino-3-hydroxyphenyl)-ethanone sulfate	1.0472	0.04233
PE(20:4(8Z,11Z,14Z,17Z)/22:0)	1.0268	0.007143
D-threonine	1.0227	0.03315

Supplementary Table S5. Organ weight

Organ-to-body weight ratio	Con	Mod	Hse	Mix	Fer
Liver (%)	2.55 ± 0.17	2.53 ± 0.25	2.58 ± 0.10	2.67 ± 0.24	2.55 ± 0.22
Kidney (%)	0.55 ± 0.05	0.62 ± 0.08	0.64 ± 0.07	0.66 ± 0.09	0.66 ±0.09
heart（%）	0.31 ± 0.04	0.37 ± 0.04	0.34 ±0.03	0.37 ± 0.03	0.34 ±0.04
Spleen (%)	0.15 ±0.01	0.16 ±003	0.14 ±0.02	0.16 ± 0.02	0.17 ±0.04

Supplementary Figure S1 Weight changes of rats.

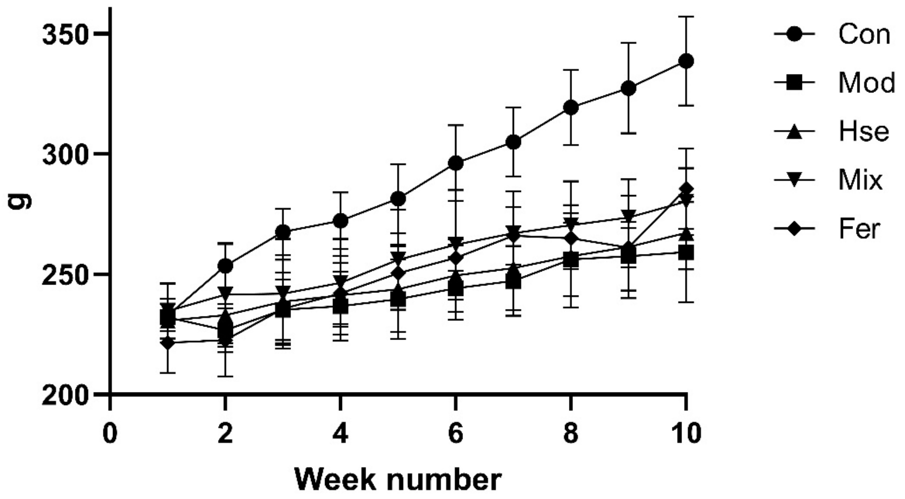

Supplementary Figure S2 Food intake of rats.

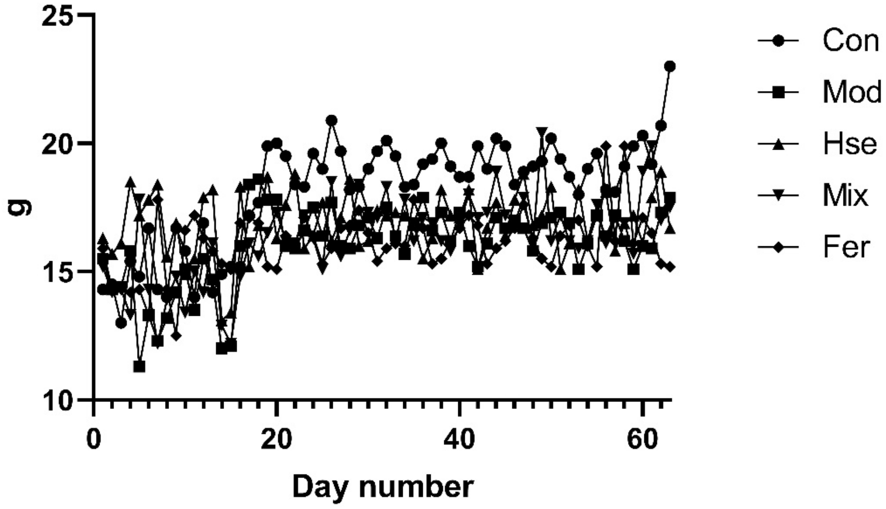

Supplement: Supplementary file 1 [file foods-13-02649-s001.zip › foods-3122402-supplementary.pdf]
